# Glutamine supplementation improves intestinal cell proliferation and stem cell differentiation in weanling mice

**DOI:** 10.29219/fnr.v62.1439

**Published:** 2018-07-23

**Authors:** Siyuan Chen, Yaoyao Xia, Guoqiang Zhu, Jiameng Yan, Chengquan Tan, Baichuan Deng, Jinping Deng, Yulong Yin, Wenkai Ren

**Affiliations:** 1 Guangdong Provincial Key Laboratory of Animal Nutrition Control, Institute of Subtropical Animal Nutrition and Feed, College of Animal Science, South China Agricultural University, Guangzhou, China; 2 Jiangsu Co-Innovation Center for Important Animal Infectious Diseases and Zoonoses, Joint International Research Laboratory of Agriculture and Agri-Product Safety of Ministry of Education of China, College of Veterinary Medicine, Yangzhou University, Yangzhou, China; 3 Laboratory of Animal Nutrition and Health and Key Laboratory of Agro-Ecology, Institute of Subtropical Agriculture, Chinese Academy of Sciences, Changsha, China

**Keywords:** glutamine, intestinal stem cells, Paneth cells, goblet cells, weaning

## Abstract

**Background:**

Intestinal stem cells can be differentiated into absorptive enterocytes and secretory cells, including Paneth cells, goblet cells, and enteroendocrine cells. Glutamine is a primary metabolic fuel of small intestinal enterocytes and is essential for the viability and growth of intestinal cells.

**Objective:**

Whether glutamine supplementation affects the differentiation of intestinal stem cells is unknown.

**Design:**

Three-week-old ICR (Institute of Cancer Research) male mice were divided randomly into two groups: 1) mice receiving a basal diet and normal drinking water and 2) mice receiving a basal diet and drinking water supplemented with glutamine. After 2 weeks, the mice were sacrificed to collect the ileum for analysis.

**Results:**

The study found that glutamine supplementation in weanling mice decreases the crypt depth in the ileum, leading to higher ratio of villus to crypt in the ileum, but promotes cell proliferation of intestinal cells and mRNA expression of Lgr5 (leucine-rich repeat-containing g-protein coupled receptor5) in the ileum. Glutamine has no effect on the number of Paneth cells and goblet cells, and the expression of markers for absorptive enterocytes, Paneth cells, goblet cells, and enteroendocrine cells.

**Conclusion:**

These findings reveal the beneficial effects of dietary glutamine supplementation to improve intestinal morphology in weanling mammals.

There are mainly two types of stem cells in the intestine: Lgr5-positive cells located at the base of crypt and Bmi1-positive cells predominantly found at the +4 position of crypt (1–3). The Lgr5^+^ stem cells are rapidly dividing stem cells and are necessary for gut renewal, while Bmi1^+^ stem cells are more quiescent and activated during stress of injury to produce intestinal progenitor cells to replace the damaged intestinal cells (4, 5). Stem cells can be differentiated into absorptive enterocytes and secretory cells, including Paneth, goblet, and enteroendocrine cells (6, 7). Paneth cells move back to the base of the crypts to intersperse between the stem cells, whereas the other types of cells migrate into the villi (6, 7). Usually, absorptive enterocytes constitute about 80% of small intestinal mucosal epithelia, while goblet cells, enteroendocrine cells, and Paneth cells take up about 5–10, 1 and 5%, respectively (8). Various parameters (e.g. intestinal microbiota, intestinal metabolites, and cellular signaling pathways) regulate the proliferation and differentiation of intestinal stem cells, leading to the alteration in the number of each type of cells and the progression of various diseases like inflammatory bowel disease, infection, and cancer (7, 9–15). Glutamine is a primary metabolic fuel of small intestinal enterocytes and is essential for the viability and growth of intestinal cells by serving as a precursor for synthesis of nucleotides, glucose, amino acids, and proteins (16, 17). However, it is unknown whether glutamine affects the intestinal stem cell differentiation.

Previous investigation showed that glutamine supplementation promotes the mRNA expression of α-defensins (a marker for Paneth cells) and C-type lectins (a marker for Paneth cells) in the jejunum and ileum in mice (18). Interestingly, glutamine supplementation promotes the expression of C-type lectins in the ileum of mice infected with enterotoxigenic *Escherichia coli* (19). These results indicate that glutamine may promote the differentiation of Paneth cells from stem cells. Besides Paneth cells, *in vitro* administration of glutamine enhances the expression of chromogranin A (a marker for enteroendocrine cells) and mucin2 (Muc2) (a marker for goblet cells) on intestinal stem cells, suggesting that glutamine may promote the differentiation of enteroendocrine and goblet cells from stem cells (20). Notably, glutamine is essential for maximal expansion of murine crypt cultures (enteroids), and glutamine deprivation induces a gradual atrophy of enteroids and decreases epithelial proliferation, while glutamine replenishment rescues proliferation of enteroid and promotes crypt regeneration (21), suggesting that glutamine may highly shape the proliferation and differentiation of intestinal stem cells. Thus, this study was conducted to uncover the influence of glutamine on the differentiation of intestinal stem cells in weanling mice. Weanling mice were selected as models because weanling mammals have a rapid renewal of intestinal cells and experience significant defects in intestinal morphology (22, 23).

## Materials and methods

### Mice

ICR (Institute of Cancer Research) male mice (3 weeks of age) were purchased from SLAC Laboratory Animal Center (Changsha, China). The mice were housed in a pathogen-free mouse colony (temperature, 25±2°C; relative humidity, 45–60%; lighting cycle, 12 h/day; 06:30–18:30 for light) and had free access to food and drinking water. Experiments in mice were conducted according to the guidelines of the Laboratory Animal Ethical Commission of the Institute of Subtropical Agriculture, Chinese Academy of Sciences, and all experimental procedures involving animals were approved by the Institute of Subtropical Agriculture.

### Glutamine supplementation for weanling mice

Three-week-old ICR male mice (without receiving any solid food before the experiment) were divided randomly into two groups (*n* = 11 for control and 12 for experimental group): 1) mice that received a basal diet (18, 24) and normal drinking water and 2) mice that received a basal diet and drinking water supplemented with glutamine (Sangon Biotech, Shangshai, China) at a dosage of 10 mg/ml. The dosage for glutamine supplementation was selected based on our previous study (25). The drinking fluid in both groups was changed daily. After 2 weeks of glutamine supplementation, the mice were sacrificed to collect the ileum after they were euthanized with CO_2_ inhalation followed by cervical dislocation to ensure death. For collection of the ileum, the middle part of the ileum samples (about 2–3 cm) was collected after phosphate-buffered saline (PBS; pH = 7.2–7.4) washing. The ileum was fixed in fresh 4% paraformaldehyde for paraffin embedding or snap frozen in liquid nitrogen for mRNA analysis. The body weights of animals were regularly monitored during the treatment period.

### Tissue histological examination

This was performed using hematoxylin and eosin (H&E) staining. Briefly, mouse ileums were fixed with 4% paraformaldehyde-PBS overnight, and then dehydrated and embedded in paraffin blocks. Sections of 5 μm were cut for histological analysis. The sections were deparaffinized and hydrated, and then stained with H&E. Villus length and crypt depth were measured using image J software. The number of goblet cells in each villus, and the number of Paneth cells in each crypt were determined. Also, immunohistochemistry against lysozyme and an Alcian blue staining were used for Paneth cell and goblet cell staining, respectively. Quantification of villus length, crypt depth, number of goblet cells, and Paneth cells were performed in at least five villi or crypts per slide. To determine the villus height, the height from the tip of the villus to the crypt opening was measured, and the associate crypt depth was measured from the base of the crypt to the level of the crypt opening. Then, the villus/crypt ratio was calculated with the ratio of villus height to relative crypt depth. Eight mice were studied from each group. The data collectors were unaware of the treatment status of the examined slides.

### Cell proliferation analysis

For cell proliferation analysis in the crypt of mouse ileum, Ki67 abundance was assessed by immunohistochemistry with anti-Ki67 antibodies (ab15580, Abcam, Cambridge; UK). Ten crypts (400×) were observed for each section. The results were expressed as the number of Ki67 positive cells in each crypt.

### RT-PCR

Total RNAwas isolated from liquid nitrogen frozen ileum using the TRIZOL regent (Invitrogen, USA) and then treated with DNase I (Invitrogen, USA) according to the manufacturer’s instructions. Synthesis of the first strand (cDNA) was performed using oligo (dT)20 and Superscript II reverse transcriptase (Invitrogen, USA). Primers were selected according to previous references (24, 26). β-actin was used as an internal control to normalize target gene transcript levels. Real-time PCR was performed according to our previous studies (18, 24).

### Statistical analyses

Data are shown as the means ± standard deviation (SD) or Standard Error of Mean (SEM). Data between two groups were analyzed by unpaired *t-*test (Prism 6.0) if the data were in Gaussian distribution and had equal variance, or by unpaired *t-*test with Welch’s correction (Prism 6.0) if the data were in Gaussian distribution but with unequal variance, or by non-parametric test (Mann–Whitney *U* test, Prism 6.0) if the data were not normally distributed (26, 27). The Gaussian distribution of data was analyzed by D’Agostino-Pearson omnibus normality test (Prism 6.0) and Kolmogorov–Smirnov test (Prism 6.0). The variance of data was analyzed by Brown–Forsythe test (Prism 6.0). Differences with *p* < 0.05 were considered significant.

## Results

### Glutamine supplementation has no effect on weight gain of weanling mice

**Fig. 1:**
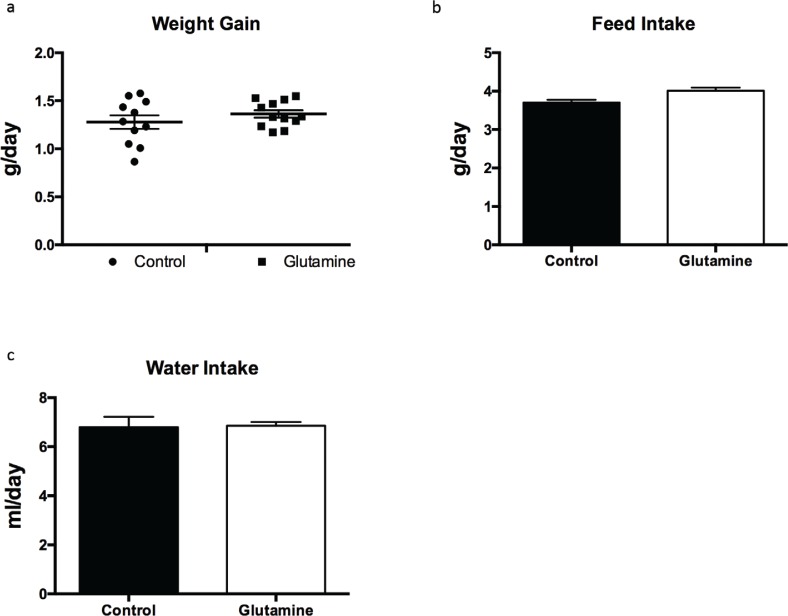
The weight gain in weanling mice. The weight gain (a), food intake (b), and water intake (c) were monitored during the experiment. Weight gain data were analyzed by unpaired *t-*test, while the data about food intake and water intake were analyzed by Mann–Whitney test. The data are Mean ± SD with an *n* = 11 in control group and 12 in glutamine group.

To explore the effect of glutamine supplementation on weanling stress, weight gain, food intake, and water intake were monitored during the experiment. Glutamine had no effect on weight gain of weanling mice (Fig. 1a), as well as the food and water intake during the experiment period (Fig. 1b, c).

### Glutamine supplementation improves the ratio of villus to crypt in the ileum of weanling mice

**Fig. 2:**
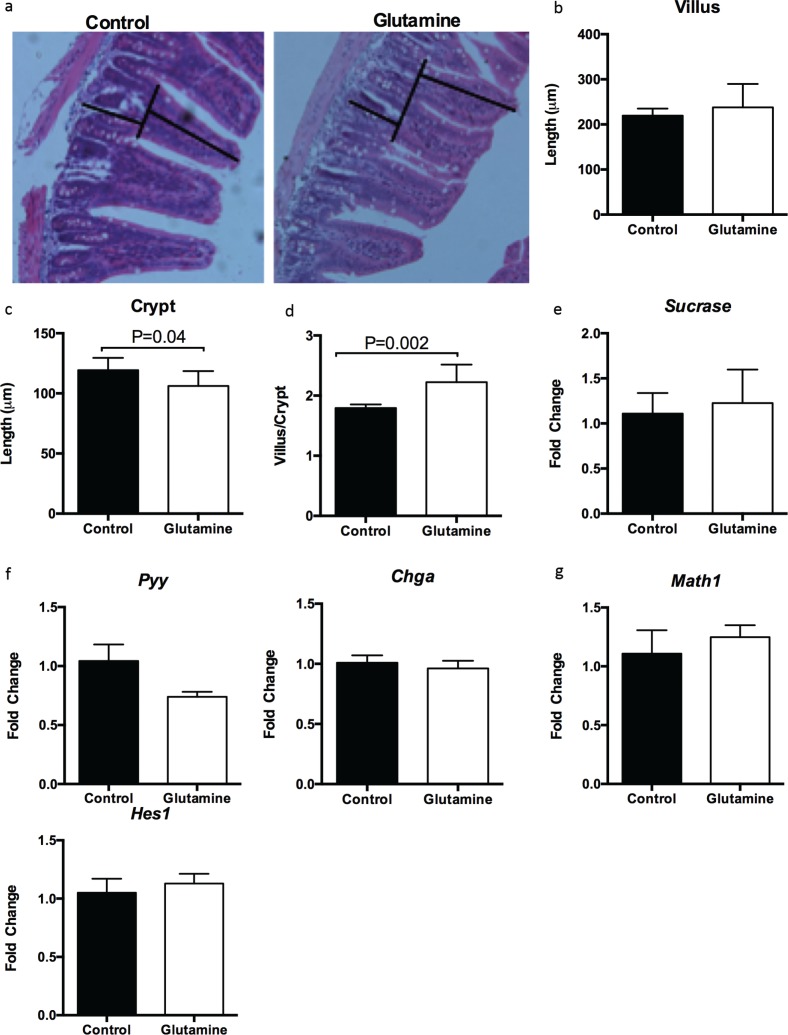
Glutamine improves the ratio of villus to crypt in the ileum of weanling mice. (a) Representative images of hematoxylin and eosin (H&E) staining in the ileum of weanling mice were shown (×200; *n* = 8). The villus length and crypt depth were measured as indicated in the image. (b–d) The statistical analysis of villus length (b), crypt depth (c), and the ratio of villus to crypt (d) from images shown at (a). The data related to villus length and crypt depth were analyzed by unpaired *t-*test, while the data related to the ratio of villus to crypt were analyzed by Mann–Whitney test. The data are Mean ± SD with an *n* = 8. (e–g) mRNA expressions of sucrase (e), enteroendocrine cells-expressed chromogranin A (CHGA) and peptide YY (PYY) (f), as well as Hes1 and Math1 (g) were analyzed in the ileum. *N* = 10, data were analyzed with unpaired *t-*test. The data are Mean±SEM.

It is well known that weanling stress induces remarkable morphological alterations in the small intestine, such as villus atrophy and crypt hyperplasia (22, 28). Glutamine had no effect on the villus length (Fig. 2a, b) but significantly decreased the crypt depth (*p* = 0.04), resulting in a higher ratio of villus to crypt in the ileum (*p* = 0.002), compared to control mice (Fig. 2a, c, d). Glutamine supplementation also had no influence on the mRNA expression of sucrase (a marker for absorptive enterocytes; Fig. 2e), enteroendocrine cells expressing chromogranin A (Chga), and peptide YY (Pyy) (Fig. 2f), as well as Hes1 and Math1 (Fig. 2g); these latter factors are known to direct intestinal epithelial differentiation into the two major lineages: the absorptive and the secretory lineage, respectively.

### Glutamine supplementation improves cell proliferation in weanling mice

**Fig. 3:**
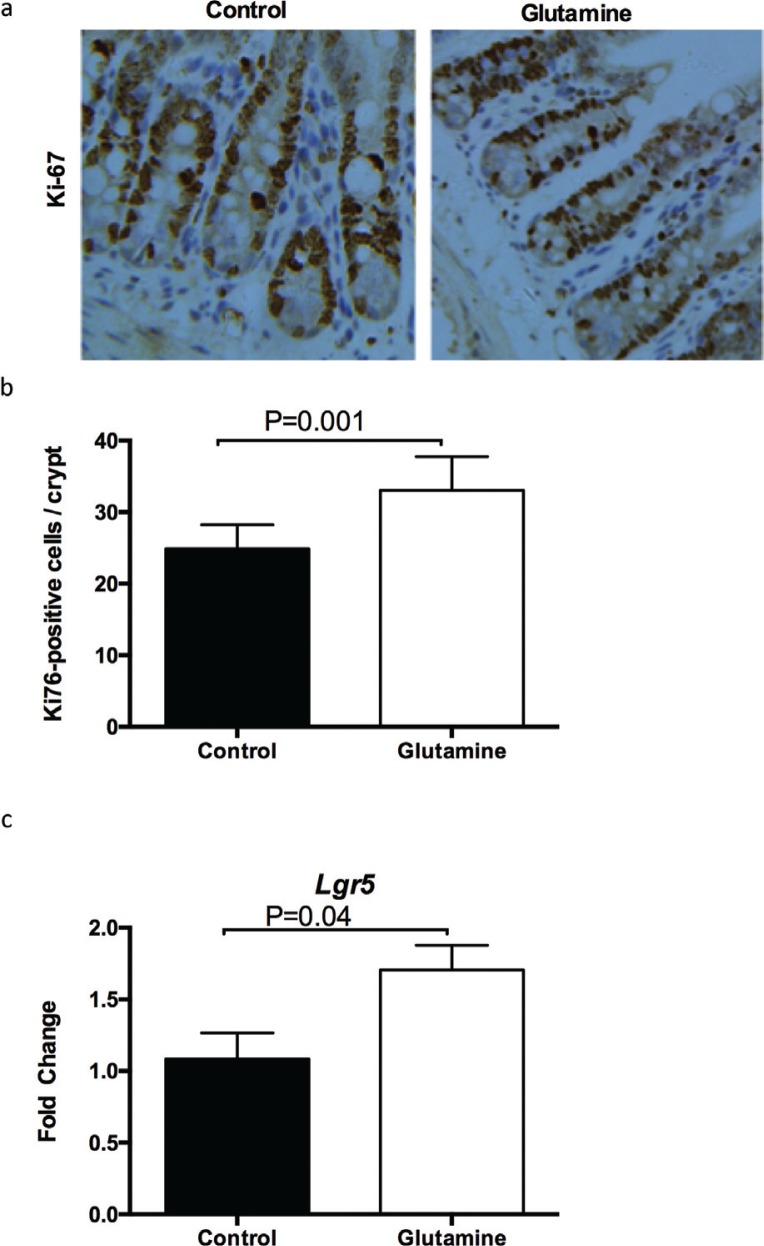
The intestinal cell proliferation after glutamine supplementation in weanling mice. (a) The representative images of immunohistochemistry (IHC) staining with Ki67 antibody in the ileum of weanling mice were shown (×100; *n* = 8). (b) The statistical analysis of Ki67-positive cells in each crypt from images shown on the (a). (c) The mRNA expression of Lgr5 in the ileum. The data were analyzed by unpaired *t-*test. The data are Mean ± SD (b) or SEM (c) with an *n* = 8 (a, b) or 10 (c).

Then, we explored cell proliferation in the ileum between glutamine-treated and control mice. Glutamine supplementation significantly (*p* = 0.001) increased the number of Ki67-positive cells in each crypt (Fig. 3a, b), suggesting that glutamine supplementation promotes the cell proliferation in the ileum of weanling mice. Similarly, glutamine supplementation enhanced the mRNA expression of Lgr5 (a marker for intestinal stem cells; Fig. 3c).

### Glutamine supplementation had no effect on the number of Paneth cells in weanling mice

**Fig. 4:**
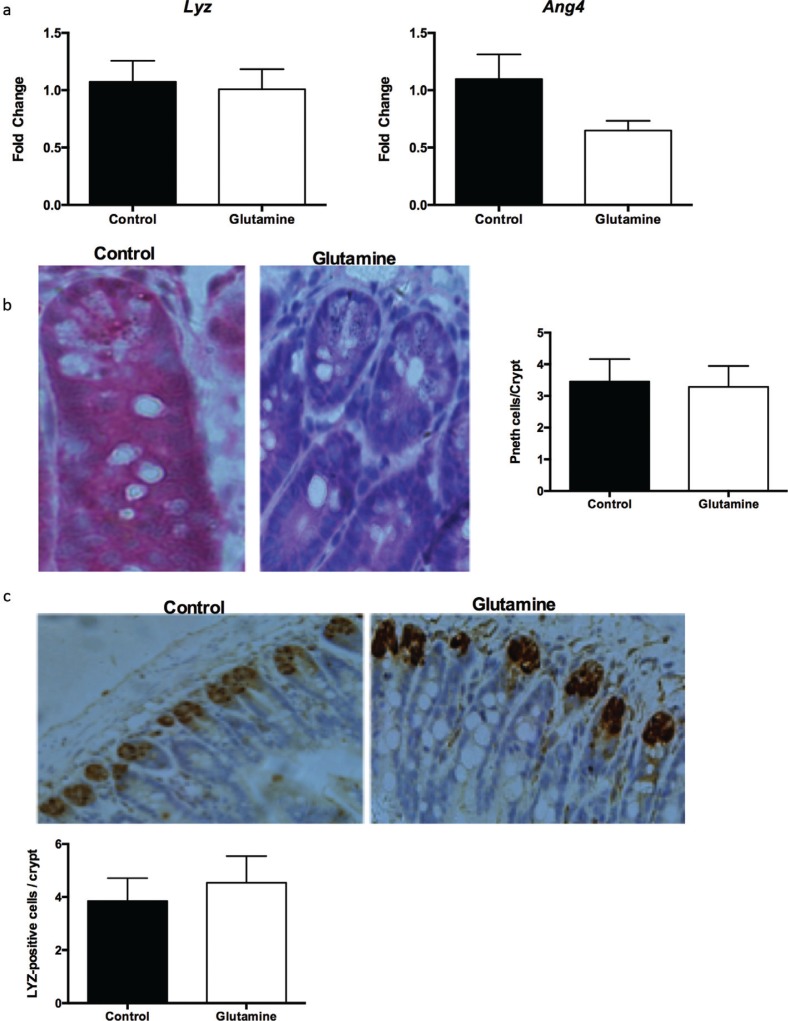
Paneth cells in the ileum after glutamine supplementation in weanling mice. (a) The mRNA expression of lysozyme (Lyz) and angiogenin 4 (Ang4) in the ileum. The data were analyzed by unpaired *t-*test. The data are Mean ± SEM with an *n* = 10. (b) The number of Paneth cells from HE staining in the ileum. Left, the representative images of Paneth cells in the ileum of weanling mice were shown (×400; *n* = 8). Right, the statistical analysis of the number of Paneth cells in each crypt from images shown on the Left. The data were analyzed by Mann–Whitney test. The data are Mean ± SD with an *n* = 8. (c) The number of Paneth cells was analyzed with lysozyme antibody. Top, the representative images of IHC staining with lysozyme antibody in the ileum of weanling mice were shown (×200; *n* = 8). Bottom, the statistical analysis of lysozyme positive cells in each crypt from images shown on the Top. The data were analyzed by unpaired *t-*test. The data are Mean ± SD with an *n* = 8.

Furthermore, we investigated the influence of glutamine on intestinal Paneth cells. There were no changes in the mRNA expression of lysozyme (Lyz) and angiogenin 4 (Ang4), which are markers of Paneth cells, in the ileum of weanling mice after glutamine supplementation (Fig. 4a). Similarly, glutamine supplementation did not influence the number of Paneth cells in the crypt of the ileum (Fig. 4b). Lysozyme staining also suggested that glutamine had no effect on the lysozyme positive cells in the crypt of the ileum (Fig. 4c).

### Glutamine supplementation had no effect on the number of goblet cells in weanling mice

**Fig. 5:**
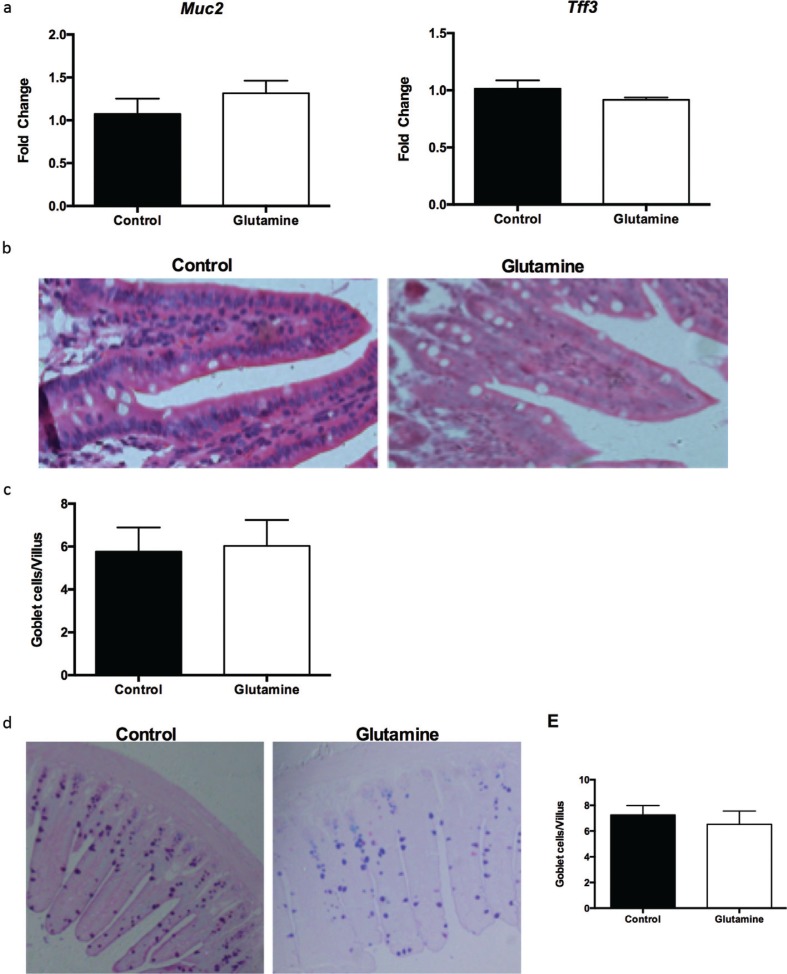
Goblet cells in the ileum after glutamine supplementation in weanling mice. (a) The mRNA expression of mucin2 (Muc2) and trefoil factor 3 (Tff3) in the ileum. The data were analyzed by unpaired *t-*test. The data are Mean ± SEM with an *n* = 10. (b, c) The number of goblet cells from HE staining in the ileum. (b) The representative images of goblet cells in the ileum of weanling mice were shown (×200; *n* = 8). (c) The statistical analysis of number of goblet cells in each villus from images shown on (b). The data were analyzed by Mann–Whitney test. The data are Mean ± SD with an *n* = 8. (d, e) The number of goblet cells was evaluated with Alcian blue staining. (d) The representative images of Alcian blue staining in the ileum of weanling mice were shown (×100; *n* = 8). (e) The statistical analysis of goblet cells in each villus from images shown on the (d). The data were analyzed using unpaired *t-*test. The data are Mean ± SD with an *n* = 8.

We also investigated the influence of glutamine on intestinal goblet cells. Glutamine supplementation had no effect on the mRNA expression of goblet cell–derived Muc2 and trefoil factor 3 (Tff3) in the ileum (Fig. 5a), as well as the number of goblet cells in the villi of the ileum (Fig. 5b, c). Alcian blue staining also demonstrated that glutamine did not change the number of goblet cells in the villi of the ileum in weanling mice (Fig. 5d, e).

## Discussion

Among the life cycle of mammals, weaning is one of the most critical periods when the infant is exposed to several alterations in nutrition, society, and environment (29–31). Thus, mammals in weanling periods suffer from serious stress responses, especially in the intestine. Increasing investigations are showing that mammals during the weanling stress experience significant defects in intestinal morphology (e.g. villus atrophy) and physiological functions (e.g. dysfunction in nutrient absorption and mucosal defensive system), and experience a shift in intestinal microbiome (e.g. increased potential pathogens) (32–35), resulting in various diseases, such as weight loss, diarrhea, and infection (26, 27). For example, our previous study found that weaning in piglets induces a reduction in villus height in the ileum, and a decrease in ratio of villus height to crypt depth in the jejunum and ileum, especially at 3 days post weaning (22). Interestingly, several functional amino acids, especially glutamine, show multiple beneficial effects on intestinal physiology (17, 36). We also showed that glutamine affects the intestinal immune system, such as the activation of toll-like receptor (TLR)-4-nuclear factor kappa B (NF-κB), mitogen-activated protein kinases (MAPK), phosphoinositide-3-kinases (PI3K)/PI3K-protein kinase B (Akt) signaling, the expression of inflammatory cytokines [e.g. interleukin (IL)-17], and the production of secretory immunoglobulin A (SIgA), leading to alteration in intestinal microbiota and inhibition of intestinal infection (18, 19, 25, 37, 38). Notably, glutamine has been shown to alleviate the weanling stress by affecting the expression of genes associated with intestinal metabolism and function (e.g. cell proliferation), resulting in alleviation in intestinal dysfunction (e.g. tight junction) and atrophy (39, 40). Similarly, this study also found that glutamine supplementation significantly decreases the crypt depth but increases the ratio of villus to crypt in the ileum of weanling mice.

In this study, glutamine had no effect on the number of Paneth cells and goblet cells, and the expression of markers for absorptive enterocytes (Sucrase), Paneth cells (Lyz and Ang 4), goblet cells (Muc2 and Tff3), and enteroendocrine cells (Chga and Pyy). This suggests that glutamine supplementation may have no effect on the differentiation of absorptive enterocytes, Paneth cells, goblet cells, and enteroendocrine cells from intestinal stem cells. This conclusion also supports the result that glutamine supplementation has no effect on the mRNA expression of Hes1 and Math1, which are known to direct intestinal epithelial differentiation into the absorptive and the secretory lineage, respectively. As far as the authors know, there is no literature about the effect of glutamine supplementation on the intestinal expression of Sucrase, Tff3, Hes1, and Math1. The result about Paneth cells is similar to our previous results which indicated that glutamine supplementation had no effect on the expression of Lyz and Ang in various mouse models (18, 19). However, it is interesting to analyze the expression of other Paneth cell–associated factors in weanling mice after glutamine supplementation because glutamine supplementation promotes the mRNA expression of other Paneth cell–associated factors (α-defensins and C-type lectins) in these previous studies (18, 19). The discovery of this study is different from a previous conclusion that glutamine enhances the expression of chromogranin A and Muc2 on intestinal stem cells *in vitro* (20). The possible explanation for this discrepancy comes from the difference between *in vivo* and *in vitro* studies. Although they found that glutamine enhances the expression of chromogranin A *in vitro*, glutamine had no effect on the expression of chromogranin A in mice (20)*.* Similarly, glutamine deprivation in murine crypt cultures does not affect the proportions of Paneth and goblet cell differentiation (21). Thus, the influence of glutamine on the differentiation of absorptive enterocytes, Paneth cells, goblet cells, and enteroendocrine cells from intestinal stem cells needs further investigations.

In this study, glutamine was found to promote the expression of Lgr5 in the ileum and the proliferation of intestinal cells in weanling mice. Similarly, glutamine promotes the expression of Lgr5 in crypt fractions isolated from small intestine of mice (20). *In vitro*, glutamine promotes the proliferation of intestinal porcine epithelial cell line J2 (IPEC-J2) (41), intestinal porcine epithelial cell line 1 (IPEC-1) (42, 43) and murine crypt cultures derived from the jejunum (21). The promotion of glutamine on intestinal cell proliferation is also observed in rats (44), mice (45), and piglets (39). However, the underlying mechanism remains to be unraveled by which glutamine promotes the proliferation of intestinal cells. Glutamine has been shown to promote the heat shock protein in the weanling piglets and in IEC-18 rat intestinal epithelial cells (46, 47), suggesting glutamine may promote the proliferation of intestinal cells through heat shock protein. Also, it remains to know whether glutamine promotes the proliferation of intestinal stem cells or other types of intestinal cells.

In conclusion, glutamine supplementation in weanling mice decreases the crypt depth, resulting in higher ratio of villus to crypt, but promotes the cell proliferation of intestinal cells in the ileum. Glutamine supplementation also promotes the mRNA expression of Lgr5 in the ileum. Glutamine has no effect on the number of Paneth cells and goblet cells, and the expression of markers for absorptive enterocytes, Paneth cells, goblet cells, and enteroendocrine cells. These findings reveal the beneficial effect of dietary glutamine supplementation in improving intestinal morphology in weanling mammals.
